# Laryngeal Mask Airway Rescue After Failed Intubation for Tracheal Resection in Severe Subglottic Stenosis: A Case Report

**DOI:** 10.7759/cureus.111388

**Published:** 2026-06-23

**Authors:** Keuntak Yuk, Hyunjeong Hwang, Seungho Kim

**Affiliations:** 1 Department of Anesthesiology and Pain Medicine, Hallym University Dongtan Sacred Heart Hospital, Hwaseong-si, KOR

**Keywords:** airway management, difficult airway, laryngeal mask airway, subglottic tracheal stenosis, tracheal resection

## Abstract

Severe subglottic stenosis poses a significant challenge during tracheal resection, particularly when conventional endotracheal intubation fails. The role of the laryngeal mask airway (LMA) as a rescue airway strategy in this setting remains poorly defined. We report the case of a 33-year-old woman with 80-90% postintubation subglottic stenosis undergoing tracheal resection. Attempts at intubation with small-caliber endotracheal tubes failed during induction. An LMA was inserted as a rescue strategy without traversing the stenotic segment. The LMA maintained adequate oxygenation with transient hypercapnia, which improved intraoperatively. Anesthesia was maintained with total intravenous anesthesia, and ventilation was performed manually. Following resection of the stenotic segment, cross-field intubation was performed, and anastomosis was completed without complications. The patient recovered without respiratory complications and was discharged on postoperative day 7. This case suggests that the LMA may serve as a practical rescue airway strategy in selected patients with severe tracheal stenosis when combined with thorough preoperative planning and well-defined backup strategies.

## Introduction

Postintubation tracheal stenosis (PITS) is one of the most frequently encountered complications of prolonged or traumatic endotracheal intubation and is the principal indication for tracheal resection with primary end-to-end anastomosis in adults [1,2]. Clinically, PITS characteristically manifests as progressive exertional dyspnea, typically emerging within weeks after extubation. Severe luminal compromise poses substantial challenges for both anesthetic induction and intraoperative airway management [2].

Subglottic stenosis refers to circumferential narrowing immediately inferior to the glottis and is regarded as severe when the luminal compromise is high-grade. Such severe narrowing markedly increases the probability of technically difficult or unsuccessful endotracheal intubation, necessitating a comprehensive and individualized airway management strategy beginning in the preoperative assessment phase [1]. Such patients present a compounded clinical challenge: an inherently elevated risk of acute airway obstruction during anesthesia induction, coupled with the difficulty of sustaining adequate alveolar ventilation throughout the operative period, rendering conventional airway techniques frequently inadequate [1,2].

We report a case of successful airway rescue using a supraglottic airway device, specifically a laryngeal mask airway (LMA), in a patient with severe postintubation subglottic stenosis who was scheduled for tracheal resection with primary end-to-end anastomosis following failed attempts at small-caliber endotracheal intubation. This report delineates the utility of the LMA as a viable rescue airway strategy in this high-acuity clinical context and contributes to the evolving literature on difficult airway management in tracheal surgery.

## Case presentation

A 33-year-old woman (height 157.8 cm, weight 76 kg) with a history of type 2 diabetes mellitus was referred for surgical management of symptomatic tracheal stenosis. The patient was admitted after a self-inflicted ligature injury, which required endotracheal intubation for airway protection. Extubation was performed two days after admission. Initial trauma evaluation excluded cervical spinal injury. Approximately one month after extubation, the patient developed progressive exertional dyspnea.

Flexible bronchoscopy revealed severe tracheal stenosis immediately inferior to the vocal cords, with an estimated reduction in the luminal cross-sectional area of 80-90% (Figure 1). Preoperative chest computed tomography (CT) confirmed subglottic stenosis at the cricoid cartilage level, with a stenotic segment measuring approximately 2 cm in length and a minimum luminal diameter of 5 mm (Figures 2, 3). This degree of narrowing was classified as Myer-Cotton grade-III subglottic stenosis. As the initial intubation and intensive care had been performed at another institution, detailed records regarding tube size, cuff pressure, and intubation conditions were not available. The thoracic surgical team planned tracheal resection with primary end-to-end anastomosis.

**Figure 1:**
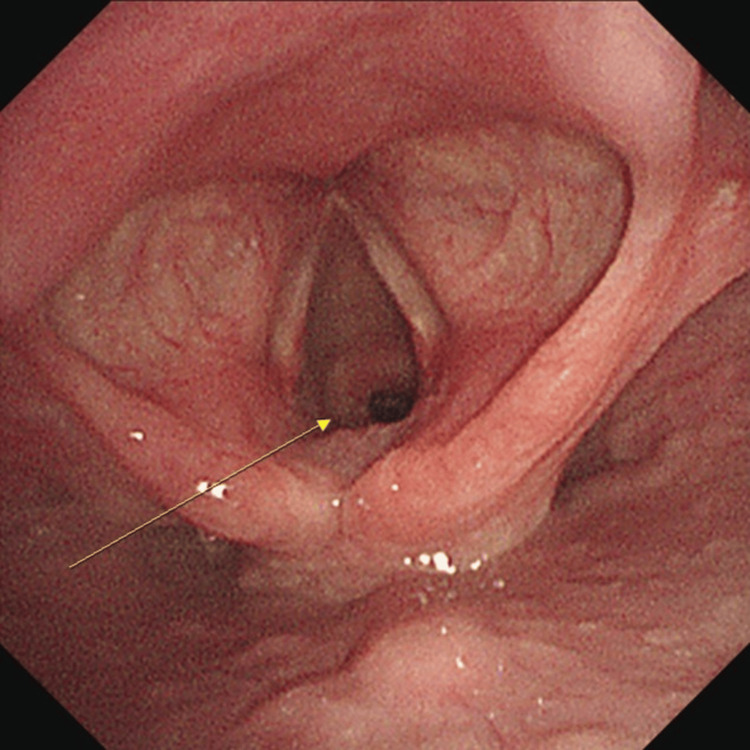
Bronchoscopic finding of severe subglottic stenosis. Bronchoscopic image showing severe subglottic stenosis with approximately 80-90% luminal narrowing located immediately below the vocal cords. The arrow indicates the site of stenosis.

**Figure 2:**
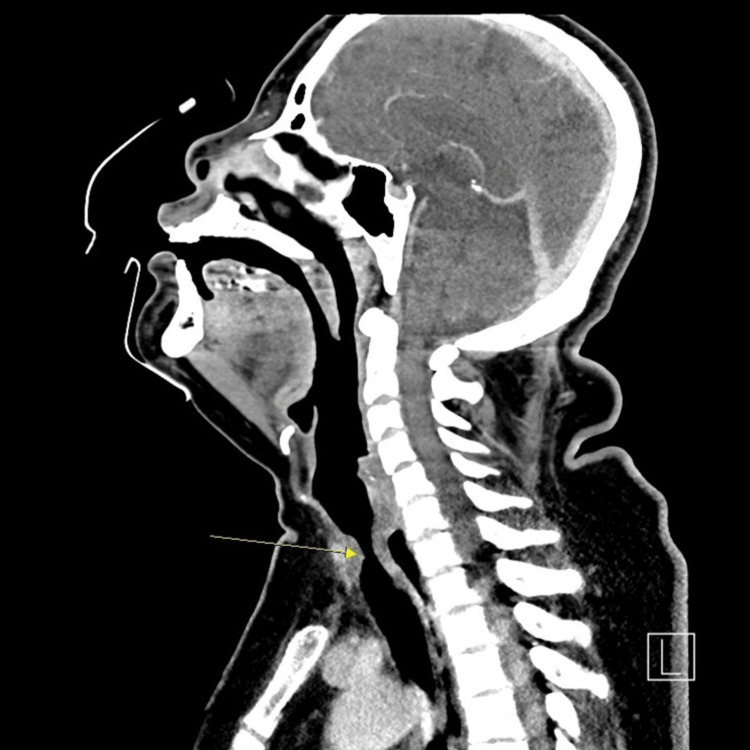
Sagittal view of the preoperative neck/chest computed tomography. The patient exhibited subglottic stenosis at the level of the cricoid cartilage, with a stenotic segment approximately 2 cm in length and a minimum luminal diameter of approximately 5 mm. The arrow indicates the site of stenosis.

**Figure 3:**
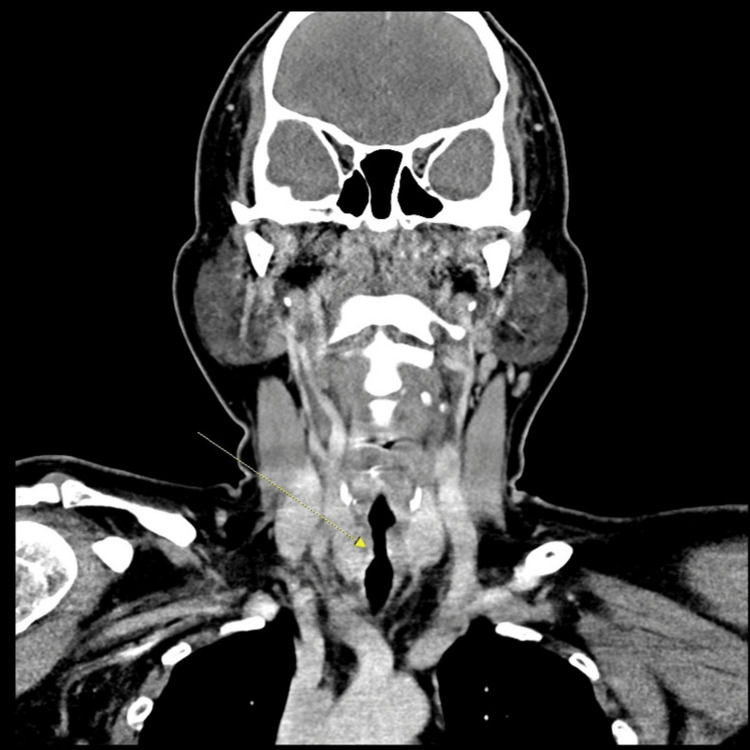
Coronal view of the preoperative neck/chest computed tomography. The patient exhibited subglottic stenosis at the level of the cricoid cartilage, with a stenotic segment approximately 2 cm in length and a minimum luminal diameter of approximately 5 mm. The arrow indicates the site of stenosis.

The principal anesthetic challenge was anticipated to be difficult airway management because the severity and subglottic location of the stenosis conferred a high probability of failure with conventional laryngoscopic intubation techniques. Preoperative arterial blood gas analysis revealed mild compensated hypercapnia (PaCO₂ 52.3 mmHg); however, the patient exhibited no respiratory distress at rest and maintained stable spontaneous ventilation. Awake fiberoptic intubation was considered the preferred initial approach; however, it was not pursued because of the patient's marked procedural anxiety and anticipated inability to cooperate. Therefore, induction was performed under general anesthesia with comprehensive preparation for sequential rescue airway strategies.

A tiered, stepwise airway algorithm was prospectively established: primary attempts with small-caliber endotracheal tubes and LMA deployment as immediate rescue options in case of failed intubation. Given the potential failure of both transglottic intubation and supraglottic ventilation, a venovenous extracorporeal membrane oxygenation (VV-ECMO) circuit and team were placed on standby in the operating room as a final salvage option.

Following the placement of a radial arterial catheter before induction, total intravenous anesthesia (TIVA) was selected to circumvent the pharmacokinetic challenges of delivering volatile anesthetic agents through a severely compromised airway. Anesthesia was induced via target-controlled infusion (TCI) of propofol and remifentanil, targeting effect-site concentrations of 3.0 µg/mL and 3.0 ng/mL, respectively. After confirming adequate bag-mask ventilation, neuromuscular blockade was achieved using intravenous (IV) rocuronium (50 mg).

In consultation with the thoracic surgical team during preoperative planning, a trial of small-caliber intubation was elected. After the onset of complete neuromuscular blockade, video laryngoscopy and intubation were attempted using a 5.0 mm internal diameter (ID) cuffed endotracheal tube; however, the tube could not be advanced through the subglottic stenosis. Subsequent attempts using a 4.5 mm ID tube were also unsuccessful. Further intubation attempts were abandoned in accordance with a pre-established airway algorithm, and an LMA Supreme (size 3) was inserted. Although weight-based sizing would conventionally indicate a larger device, a size 3 was chosen as the initial rescue device to facilitate prompt, atraumatic placement. Ventilation through the LMA was nonetheless clinically adequate, providing stable gas exchange without a significant oropharyngeal leak and without the need for passage of an airway device through the stenotic segment. Airway seal (leak) pressure was not formally measured. Subsequently, a central venous catheter was placed in the right subclavian vein.

Arterial blood gas analysis obtained immediately following LMA placement revealed pH 7.269, PaCO₂ 76.3 mmHg, PaO₂ 437.1 mmHg, and SaO₂ 100%. Although significant hypercapnia was observed, the arterial oxygenation remained well-preserved, and the patient remained hemodynamically stable (blood pressure approximately 100/60 mmHg, heart rate 70-80 beats/min, no arrhythmias, and no vasoactive support), consistent with transient permissive hypercapnia. Repeat analysis at the time of surgical incision demonstrated progressive improvement in ventilatory parameters: pH 7.296, PaCO₂ 63.2 mmHg, PaO₂ 513.1 mmHg, and SaO₂ 100%.

Manual pressure-controlled ventilation was used throughout the initial operative phase instead of volume-controlled mechanical ventilation, enabling continuous real-time adjustment of the tidal volume and inspiratory timing in response to surgical manipulation. Tidal volumes were maintained between 300 and 450 mL, with peak airway pressures of 15-21 cmH₂O. A fraction of inspired oxygen (FiO₂) of 1.0 was maintained during the initial resection phase.

Following resection of the stenotic segment, a 7.0 mm ID endotracheal tube was inserted into the distal tracheal stump under direct surgical visualization, and volume-controlled mechanical ventilation was initiated with FiO₂ reduced to 0.6-0.7. During the anastomotic phase, intermittent apneic intervals were required to facilitate optimal surgical exposure. Each apneic interval was limited to less than 30 seconds through prospective coordination with the surgical team, and oxygen saturation was maintained at 95% or above throughout these periods. As anastomotic completion approached, the distal endotracheal tube was withdrawn, and manual ventilation via the LMA was resumed.

Arterial blood gas analysis following completion of the anastomosis demonstrated normalization of gas exchange: pH 7.405, PaCO₂ 45.3 mmHg, PaO₂ 446.3 mmHg, and SaO₂ 100%. Despite the intermittent apneic intervals required during anastomosis, no episode of clinically significant hypoxemia occurred, and the transient hypercapnia observed earlier did not recur or persist following normalization of gas exchange.

After the surgery, the neuromuscular blockade was reversed with sugammadex (200 mg). After confirming complete spontaneous ventilatory recovery and a train-of-four (TOF) ratio of 1.0 (100%), the LMA was removed using direct laryngoscopic visualization. There was no evidence of vocal cord dysfunction or paralysis, and the patient's respiratory status remained stable throughout the emergence period. The total operative time was 1 hour and 50 minutes. Ventilation was maintained via the LMA for approximately 50 minutes prior to resection, and cross-field ventilation through the distal endotracheal tube was used for approximately 25-30 minutes during the resection and anastomosis.

The patient was subsequently transferred to the intensive care unit (ICU) and received supplemental oxygen via a simple face mask. The postoperative course was uneventful. Flexible bronchoscopy performed on postoperative day 4 demonstrated a patent airway with an intact anastomosis and no evidence of restenosis. The patient was discharged on postoperative day 7. She was subsequently reviewed in the outpatient clinic at two weeks and at one month postoperatively, remaining free of dyspnea or other respiratory symptoms, with no clinical evidence of recurrent stenosis. A follow-up CT was not obtained, as the patient remained clinically asymptomatic. Informed consent was obtained from the patient for publication of this case report and its accompanying images.

## Discussion

Tracheal resection with primary end-to-end anastomosis for severe subglottic stenosis is one of the most demanding scenarios in perioperative airway management. The anesthesiologist must concurrently ensure a secure airway, maintain adequate alveolar gas exchange, and provide an unobstructed operative field throughout the procedure, during which airway continuity is deliberately interrupted. No single airway management strategy is universally optimal; each technique carries inherent advantages and limitations, and the present case illustrates how a staged, geometry-driven plan, rather than any single device, can reconcile these competing demands [3].

Airway management strategies for tracheal resection include small-caliber endotracheal intubation, supraglottic airway devices (SADs), intraoperative cross-field ventilation, high-frequency jet ventilation (HFJV), and extracorporeal circulatory support such as cardiopulmonary bypass or ECMO [2-4]. A systematic review by Schieren et al. reported that, although conventional transglottic techniques remain the most frequently used, alternative approaches, including SADs and ECMO, can achieve acceptably low failure rates when applied with rigorous patient selection and well-defined contingency strategies [4].

Transglottic intubation with an appropriately sized tube, selected from preoperative cross-sectional imaging, remains the most reliable means of definitive airway control and is a legitimate first-line strategy whenever the measured lumen will accommodate a tube of adequate caliber. When the stenotic diameter is known, a small, well-lubricated tube can often be advanced atraumatically to or beyond the lesion under direct or video-laryngoscopic vision, securing a cuff-protected distal airway. Its limitations, however, are dictated by geometry: as the lumen approaches a critical diameter, the external diameter of a tube long enough to sit securely beyond the stenosis may exceed the available lumen, and forced passage risks mucosal edema, hemorrhage, and conversion of a high-grade stenosis into complete obstruction [1]. Small-caliber tubes also require higher airway pressures to deliver adequate tidal volumes, and an indwelling transglottic tube may encroach on the operative field and transmit mechanical stress to the fresh anastomosis [3,5]. In our patient, whose minimum luminal diameter measured approximately 5 mm, the constraints of external diameter and length could not be reconciled. Cuffed tubes of 5.0 and 4.5 mm internal diameter, with external diameters of approximately 6.9 and 6.2 mm, clearly exceeded the lumen and could not be advanced despite generous lubrication. A 4.0 mm tube was not attempted: its external diameter (approximately 5.5 mm) still marginally exceeded the lumen, and its length would not have permitted stable positioning beyond the lesion. An uncuffed pediatric tube narrow enough to traverse a 5 mm lumen would likewise have been too short to sit securely beyond the stenosis and would have forfeited cuff protection against aspiration. In consultation with the surgical team, we therefore withheld further attempts, judging that the risk of precipitating complete airway loss through repeated instrumentation outweighed the marginal benefit of persisting [1].

Several alternative approaches were also weighed during preoperative planning. Awake fiberoptic intubation, frequently advocated as the preferred primary strategy in critical subglottic stenosis [3], was precluded by the patient's marked procedural anxiety and anticipated inability to cooperate, which carried its own risk of agitation and acute airway compromise; the same luminal geometry, moreover, would have constrained transglottic tube placement whether awake or under anesthesia. Rigid bronchoscopy is a mainstay of endoscopic airway management and can provide a ventilation channel; its use as a primary strategy, however, depends on dedicated equipment and operator expertise, and the technique is better suited to endoscopic dilation than to the open resection planned in this patient. An emergency surgical airway was similarly constrained: cricothyrotomy was precluded by the cricoid-level location of the stenosis, and a tracheostomy below the lesion would have encroached on the planned resection field.

Jet ventilation, whether delivered as low-frequency Sanders injection or as HFJV, is a long-established technique for airway surgery and provides a largely unobstructed operative field. It has been used for the resection of high tracheal and subglottic stenoses, in some series combined with a supraglottic airway across successive operative phases [6-8]. The principal concern in a fixed severe stenosis is expiratory: gas that enters the airway must leave through the same narrowed segment, and if expiration is incomplete, breath-stacking, dynamic hyperinflation, auto-PEEP (positive end-expiratory pressure), and barotrauma may ensue. This expiratory-egress constraint, however, is not specific to jet ventilation; it is an intrinsic property of any positive-pressure ventilation applied above a fixed stenosis, including the LMA-based manual ventilation used in our patient, in whom it manifested as transient hypercapnia. The constraint is generally manageable with deliberate technique: low respiratory rate, low inflation or driving pressure, prolonged expiratory time, and continuous confirmation of chest deflation. High-frequency variants, which shorten the available expiratory interval, warrant correspondingly greater vigilance. In a systematic review of endoscopic airway stenosis management, jet ventilation was associated with a higher technique-failure rate (approximately 30%) but a low incidence of intraoperative hypoxia (around 2%), comparable to that observed with supraglottic airways [9]. In our institution, jet ventilation was not selected because the equipment and procedural expertise required for its safe use were not available; we therefore regard its non-use as an institutional limitation rather than a physiological contraindication, and centers experienced in these techniques may reasonably adopt jet ventilation as a primary strategy in comparable cases.

In our patient, ventilation was established and maintained with an LMA after small-caliber intubation had failed. Crucially, a supraglottic airway does not bypass or relieve the stenosis: inspired and expired gas must still traverse the narrowed segment. The lumen, though impassable to an adequately sized tube, remained patent to ventilatory flow, so ventilation could be sustained at the cost of a degree of hypercapnia -- an expected consequence rather than a sign of device failure. The value of the LMA in this setting lies elsewhere, in what it avoids. By ventilating proximal to the lesion, it spares the critical segment mucosal edema, hemorrhage, and the risk of complete obstruction that attends repeated transglottic instrumentation [6,7], and it leaves the surgical field unobstructed, an advantage when the stenosis lies immediately below the glottis and an indwelling tube would interfere with placement of the anastomotic sutures [4,8,10]. It can also be deployed rapidly and atraumatically at the moment intubation is abandoned.

The hypercapnia was, as anticipated, transient: it resolved without hemodynamic consequence as CO₂ cleared, while oxygenation remained complete throughout. Zhu et al. similarly reported that LMA ventilation preserved oxygenation, with transient hypercapnia and no significant complications, in selected patients with postintubation stenosis [11]. In a single-center retrospective cohort, Adiyeke et al. found LMA-based management to be associated with a lower rate of intubated transfer to the ICU, a shorter ICU stay, and fewer positive tracheal cultures than orotracheal intubation, with comparable postoperative mortality [12]; while encouraging, these observations derive from a nonrandomized series and should not be overgeneralized.

The limitations of an LMA in this context must be acknowledged. Recognized risks include inadequate minute ventilation, aspiration of gastric contents, and difficulty sustaining gas exchange when airway resistance is high [3]. Ventilatory adequacy may be compromised if resistance rises acutely or high inflation pressures become necessary, and ventilatory efficiency may likewise deteriorate during abrupt surgical manipulation of the airway or unanticipated changes in patient position [4,8]. Hypercapnia, in particular, may be more pronounced and clinically consequential in patients with limited cardiopulmonary reserve or raised intracranial pressure, in whom permissive hypercapnia may not be tolerated.

Extracorporeal support -- VV-ECMO or veno-arterial ECMO, and historically cardiopulmonary bypass -- provides oxygenation and carbon dioxide removal independent of the airway and is the definitive fallback when ventilation cannot be established by any luminal route [3,4]. It is, however, the most invasive of the available options, requiring vascular cannulation and systemic anticoagulation, with attendant risks of bleeding (of particular concern in the surgical field) and vascular injury, as well as considerable resource demands. For these reasons, it is reserved for carefully selected patients, or for situations in which less invasive techniques are likely to prove inadequate, rather than adopted as a routine first choice [4,11]. In our patient, a VV-ECMO circuit and team were placed on standby before induction. Preparing extracorporeal rescue as a planned contingency, rather than improvising it as an emergent last resort, widened the safety margin and allowed the less invasive airway strategy to be pursued with confidence.

Several considerations favored TIVA for the maintenance of anesthesia. The delivery of inhalational agents is inherently unreliable when ventilation is intermittently disrupted by elevated airway resistance and by the planned apneic intervals of tracheal anastomosis. Repeated transitions between apnea and resumed ventilation produce large fluctuations in end-tidal anesthetic concentration, rendering titration of volatile agents imprecise and making stable anesthetic depth difficult to maintain. Wright et al. documented a progressive institutional shift from inhalational anesthesia to TIVA in a large series of patients with PITS [2], and contemporary reviews advocate TIVA as the preferred technique for tracheal resection, citing its ventilation-independent pharmacological consistency [3,5].

Manual ventilation, rather than volume-controlled mechanical ventilation, was used through the LMA. In severe tracheal stenosis, the dominant impairment is expiratory flow limitation at the stenotic segment, which predisposes patients to dynamic air trapping distal to it and to auto-PEEP [1]. Mechanical ventilation with fixed respiratory rates and inspiratory-to-expiratory ratios risks excessive peak airway pressures or insufficient expiratory time when resistance rises acutely, whereas manual ventilation provides continuous tactile feedback on compliance and resistance, allowing immediate adjustment of inspiratory pressure, tidal volume, and expiratory time. Isono et al. cautioned specifically against dynamic hyperinflation during controlled ventilation in severe tracheal stenosis and stressed individualized titration of ventilatory pressure and expiratory time [1]. Zhu et al. and a recent review likewise advocated gentle or manual ventilation during the open-airway phase [5,11].

The instructive value of this case lies not in the device that succeeded but in the framework that made its use safe. Severe subglottic stenosis admits no single, universally applicable technique: the choice among a sized transglottic tube, jet ventilation, a supraglottic airway, and extracorporeal support is dictated by the measured geometry of the lesion and by the equipment and expertise available locally [3,4]. Moreover, apart from a cuffed tube seated beyond the lesion, every technique -- supraglottic ventilation and jet ventilation alike -- requires expired gas to egress through the narrowed segment, so that some degree of hypercapnia should be anticipated rather than interpreted as failure. What protected this patient was a tiered plan formulated before induction and matched to that patient's physiology: cross-sectional imaging defined the lesion; a sized tube was attempted first and abandoned when geometry precluded it; the LMA served as the immediate rescue when the stenosis could not be traversed; cross-field intubation provided distal control once the airway was open, and VV-ECMO stood ready throughout as the final escalation. The LMA was not inherently superior to the alternatives; it was the rung that fit this patient's anatomy and our institution's capabilities; in a different geometry, or in a center experienced with jet techniques, a correctly sized tube or jet ventilation might equally have been the rational first choice. The transferable lesson is therefore procedural rather than device-specific: assess the geometry, predefine a stepwise algorithm in which each rung is chosen for a physiological reason and backed by an explicit fallback, and anticipate the shared limitations of whichever technique is selected.

## Conclusions

In conclusion, this case suggests that the LMA, combined with TIVA and manual ventilation, can serve as a practical rescue option in carefully selected patients with severe subglottic stenosis undergoing tracheal resection, even after failed endotracheal intubation. Transient permissive hypercapnia occurred during LMA ventilation and resolved as gas exchange normalized, underscoring the need for vigilant arterial blood gas and hemodynamic monitoring. The approach is not a universal solution; its success depends on careful patient selection, preserved oxygenation above the stenosis, a cohesive multidisciplinary team, and a predefined tiered backup plan, up to and including extracorporeal support.
